# Improvement of physico-chemical properties of dextran-chitosan composite scaffolds by addition of nano-hydroxyapatite

**DOI:** 10.1038/s41598-018-30720-2

**Published:** 2018-08-15

**Authors:** Emad El-Meliegy, N. I. Abu-Elsaad, Abeer M. El-Kady, Manar A. Ibrahim

**Affiliations:** 1 Department of ceramics, National research Centre, El-Tahrir street, Dokki, Cairo, Egypt; 2 Department of Physics, Faculty of Science, Zagazig University, Zagazig, Egypt; 3 Department of glass, National research Centre, El-Tahrir street, Dokki, Cairo, Egypt

## Abstract

Nano-hydroxyapatite was incorporated into polymer matrix of Dextran/Chitosan to achieve a novel composite scaffold by freeze drying technique. The synthesized composite scaffolds were recognized by different performances such as: X-ray diffraction (XRD), Fourier transform infrared spectroscopy (FT-IR) and Scanning electron microscope (SEM). The results revealed the complex formation between dextran and chitosan with an excellent dispersion of nHA inside the polymer matrix. The SEM images showed the presence of interconnected pore structure inside the scaffolds. The porosity of the composites was found to decrease from 82% to 67% by adding nanohydroxyapatite to the polymer matrix of Dextran/Chitosan. The mechanical properties of the scaffolds were measured by compression test. The obtained results verified that the presence of nHA can noticeably enhance young’s modulus and compressive strength of the composite scaffolds. All the obtained results essentially recommend that these composites can be a good candidate for bone tissue engineering applications.

## Introduction

Recently, nanocomposites composed of biopolymeric matrices and bioceramic fillers are considered as attractive alternatives for conventional materials (e.g., autografts, allografts, and metallic orthopedic biomaterials) to be used as scaffolds for transplantation of bone reconstruction. A perfect scaffold is described by controllable biodegradability, excellent biocompatibility, cytocompatibility, appropriate microstructure and mechanical properties^1,2^.

Dextran (Dex) is a neutral, hydrophilic, biocompatible and biodegradable polysaccharide with an α-(1,6) pyranose ring linkage, that is formed by different bacterial strains from sucrose via the effect of dextransucrose enzyme. These properties as well as its biochemical symmetry with the ECM make it suitable for using as scaffolds in tissue engineering. It is characterized by its hydrophilic nature and inability to supply a surface that supports cell adhesion and spreading, which is essential for cell production and osteogenesis in bone tissue engineering applications^3^.

The natural biopolymer chitosan is recently a subject of interest in tissue engineering^4,5^. Chitosan (CS) is a naturalistic polymer comprising glucosamine and N-acetylglucosamine resulted from the deacetylation of chitin^6^. Since it is degraded by the enzymes of human body, creating non-toxic side products, it is extensively applied in tissue engineering concepts^7^. Furthermore, chitosan has antimicrobial, hemostatic and osteoconductive properties, which make it appropriate for engineering hard tissues, but its mechanical properties and biological performance should be improved. The mechanical properties of the scaffold are essential for hard tissue like bone to diffuse mechanical force and produce matrix mineralization.

There are many reports in the literature on the composite scaffolds for tissue regeneration is utilizing both chitosan and hydroxyapatite as basic materials. Madihally *et al*.^1^ equipped porous chitosan scaffolds with excellent microstructure in several tissue-relevant geometries by freeze-drying method. Kashiwazaki *et al*.^8^ prepared porous chitosan/hydroxyapatite (HA) nanocomposites scaffold by the co-precipitation and porogen (NaCl) leaching method (60–87% porosity, pore diameters 100–200 μm). Tsiourvas *et al*.^9^ investigated the structural and mechanical properties of hydroxyapatite/chitosan-based porous three-dimensional scaffolds with complex geometries. However, up to date, there is still no research on the preparation of a Dex-CS/nHA composite scaffold. Hence, here we first combined nano-hydroxyapatite (nHA) into Dex-CS to achieve a novel composite scaffold of Dex-CS/nHA by freeze-drying method.

In this paper, we investigate the preparation of Dex-CS/nHA composite scaffolds with different weight percentage of nHA of (0, 20, 30 and 40 Wt%). Also, the influence of nHA on the morphological and mechanical properties of the scaffolds is studied and compared.

## Materials and Methods

### Materials

Calcium nitrate tetrahydrate [Ca(NO_3_)_2_.4H_2_O] was purchased from Oxford Mumbai, India. Di-ammonium hydrogen orthophosphate [(NH_4_)_2_HPO_4_], sodium hydroxide (NaOH), ethanol and acetic acid were obtained from El-Naser pharmaceutical chemicals company (Adwic-Egypt). Chitosan [(C_6_H_10_NO_4_)_n_] and Dextran [(C_6_H_10_O_5_)_n_] were purchased from Sigma Aldrich.

### Preparation of Hydroxyapatite (HA)

Nano-hydroxyapatite (nHA) was prepared by solution-precipitation method using calcium nitrate tetrahydrate [Ca(NO_3_)_2_·4H_2_O] and di-ammonium hydrogen orthophosphate [(NH_4_)_2_HPO_4_] as sources of calcium (Ca) and phosphorous (P) ions, respectively.

A suspension of 1 M Ca(NO_3_)_2_·4H_2_O was prepared. The Ca:P ratio for stoichiometric HA = 1.67. Keeping that ratio constant, a suspension of 0.6 M (NH_4_)_2_HPO_4_ was organized by dissolving (NH_4_)_2_HPO_4_ in distilled water. A 1 M calcium salt solution was dropped slowly from a burette into a stirred solution of 0.6 M aqueous phosphate solution with heating up to 60 °C on a hot plate. Then NaOH solution was added dropwise to control the pH of the solution mixture at ≈11 during the preparation process keeping the solution to stirred for 3 hours. The obtained suspension was aged at room temperature for 5 days. The HA precipitates were filtered from the solution, washed with distilled water and dried in a furnace at 60 °C. Finally, the obtained powders were sintered at 1050 °C for 1 hr then grounded to fine particles^10^.

### Preparation of the Composite Scaffold

Dextran-Chitosan composite scaffold was synthesized via blending method. A dextran-chitosan aqueous solution of 8 Wt% was prepared by dissolving dextran and chitosan powders into acidified distilled water (3 Wt% acetic acid) with constant stirring overnight at room temperature to get a homogeneous mixture. Then the mixture was moulded, frozen to freeze the solvent and lyophilized in a freeze-dryer at −90 °C for 48 h and a porous scaffold was achieved. This sample was denoted by A. Then, HA nanoparticles (nHA) powder was added with different proportions into a dextran-chitosan aqueous solution of 3 Wt% acetic acid (20, 30 and 40 Wt%) with constant stirring overnight at room temperature to get a homogeneous mixture. After that, the mixture was moulded, frozen to freeze the solvent and lyophilized in a freeze-dryer (SCANVAC, Denmark) at −90 °C for 48 h to get porous scaffolds. These samples were symbolized by B, C and D, respectively.

### Characterization of the scaffolds

#### X-ray diffraction (XRD)

X-ray diffraction (XRD) measurements were aquired using a diffractometer model (Type PHILIPS X’ Pert Diffractometer) having a Cukα (λ = 0.154056 nm) at room temperature. The diffraction patterns were recorded automatically with scanning speed (4°/min) and chart speed (20 mm/min). The X-ray diffraction patterns were investigated in the range of 2θ between 4° and 60°.

#### Fourier transform infrared spectroscopy (FTIR)

FT-IR spectra of dextran, chitosan and all composite scaffolds (A, B, C and D) were obtained using an FT-IR spectrometer model (FT/IR-6100 type A).The scaffolds were grounded into fine powder. Then, the obtained powder was mixed with KBr powder and compacted into pellets for FT-IR examination. The spectra were investigated in the wave number range of 400–4000 cm^−1^.

#### Scanning electron microscopy (SEM)

The morphologies of the synthesized scaffolds (A, B, C and D) were studied by scanning electron microscope (SEM). Scaffolds were cut into thin sections with a razor. After being coated with gold in a sputtering device, the samples were examined with a scanning electron microscope (QANTA FEG 250).

#### Porosity and density

The total porosity (P) and density of the prepared scaffolds were measured using a liquid displacement method^11^. Scaffolds with a given dry weight were putted in absolute ethanol for 24 h. Ethanol was used because it can penetrate through the scaffolds without bulging or shrinking the matrix and the scaffolds would not be dissolved in ethanol under room temperature^12^. The total amount of ethanol that the scaffolds were capable to absorb during 24 h was estimated by utilizing the following relationship:

1 $${\rm{P}}( \% )=\frac{{{\rm{W}}}_{2}-{{\rm{W}}}_{1}}{\rho V}$$

where W_1_ and W_2_ indicate the weight of the scaffolds before and after submerging in ethanol, respectively, V is the volume of the scaffold before submerging and ρ is a constant density of ethanol.

#### Mechanical Properties

The mechanical properties of the composite scaffolds were evaluated by subjecting the samples to the compression test using a computer-controlled Universal Testing Machine (LloyD- LR 10 K, England). The compression (mm) and load (N) were assessed at a crosshead speed of 2 mm/min at room temperature. The compressive elastic moduli were estimated as the tangent slope of the stress-strain curves in the initial linear region of the compression curve. The compressive strength of the samples was obtained by dividing the maximum force by the initial cross-sectional area. Three specimens were measured for each composite and the average values and standard deviations were recorded.

## Results and Discussion

### X-ray diffraction (XRD)

**Figure 1:**
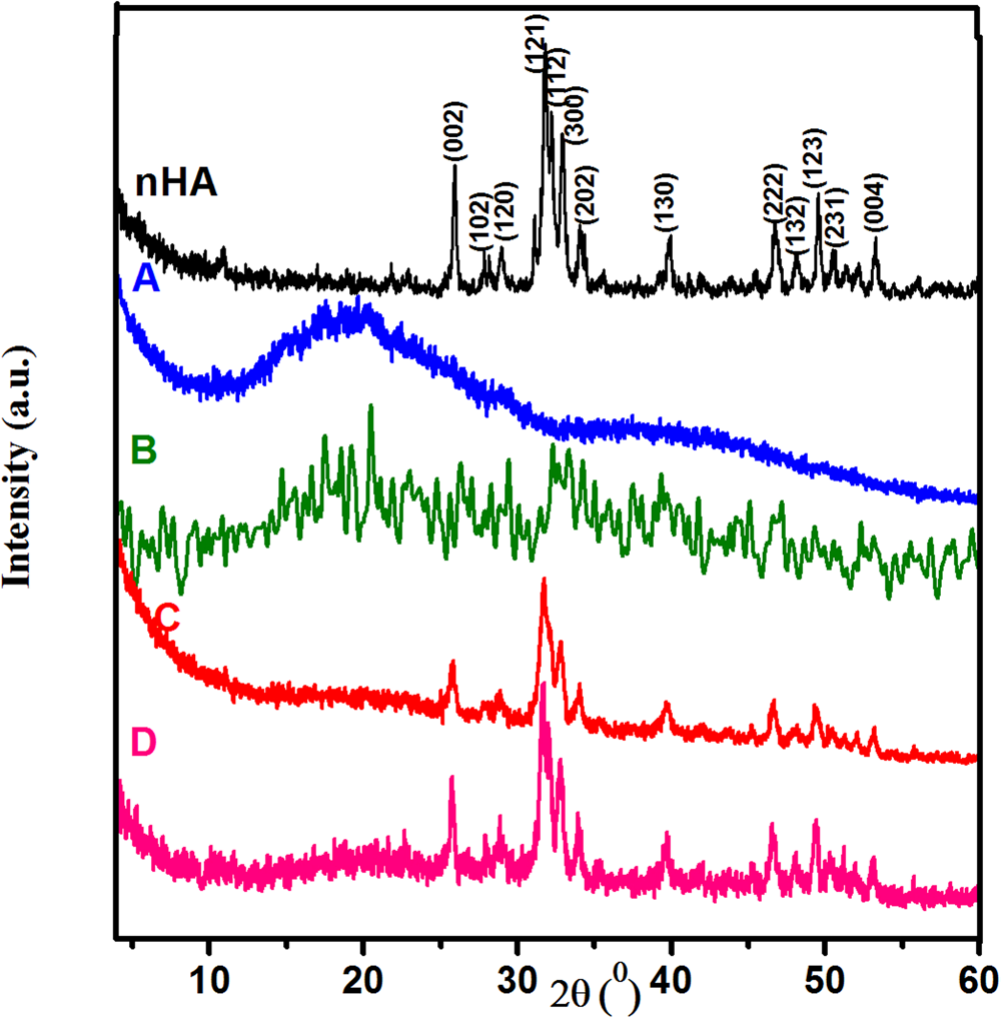
XRD patterns for nHA and Dex-CS/nHA scaffolds with different concentrations of HA (A) 0, (B) 20, (C) 30 and (D) 40 Wt%.

The room temperature XRD patterns of nHA and composite scaffolds A, B, C and D are displayed in Fig. 1. The XRD pattern of HA nanoparticles reveals the existence of the peaks which characterize the HA at 25.92°, 28.95°, 31.79°, 32.24°, 32.89°, 46.71°, 49.53° and 53.27° corresponding to the diffraction planes (002), (120), (121), (112), (300), (222), (123) and (004), respectively. This indicates the formation of HA in the crystalline structure. The structure can be indexed as a hexagonal crystal system of space group P6_3_/m with ICDD card (No.01-080-7086). Meanwhile, the pattern of the composite scaffold A (dextran/chitosan) shows two broad diffraction peaks centered at 2θ of 20.2° and 39.39°, which indicates its semi-crystalline nature. These two peaks can be attributed to the presence of CS and the interaction of Dex and CS, respectively.

When nHA particles added to Dex-CS composite with different proportions (20, 30 and 40 Wt%), the crystalline features of the constituents HA and Dex-CS still exists, as verified from the existence of their respective characteristic crystalline peaks. Moreover, XRD pattern of the composite B shows the two characteristics peaks of Dex-CS composite at 2θ of 20.4° and 39.37° and the high intensity diffracted peak of nHA at 32.21°, which suggests that the obtained composite has low crystallinity. As the amount of nHA percentage increases (composites C and D), an increasable change in both crystallinity and the intensity of the reflection planes has been observed in the respective XRD pattern. It is worthy to note that the crystallinity of the Dex-CS/nHA composites scaffolds is lower than pure nHA, which can be attributed to the occurrence of the polymer matrix^13^.

The broadening of the diffraction peaks verified the nanocrystalline characteristics of the samples. The peak broadening of XRD pattern can be used to determine the crystallite size (L) in the perpendicular direction of the crystallographic plane based on Scherrer’s formula^14,15^.

2 $$L=\frac{K{\rm{\lambda }}}{{\rm{\beta }}\,\cos \,{\rm{\theta }}}$$

where K is a constant related to the crystallite shape (K ≈ 1), λ is the wavelength of X-ray radiation (0.154056 nm), β is the full width of the high intensity peak at half of its maximum intensity (rad). The average values of crystallite sizes of all composites lie in the range of 3–33 nm as tabulated in Table 1. It could be observed that the estimated crystallite size of composite with high concentration of nHA (composite D) is ~23 which is analogous to the crystallite size of bioapatite in bone tissue. The average size of bone apatite is normally ~20 nm, which was evidenced by different experimental techniques^16^.

**Table 1:** Crystallite size (L) and crystallographic parameters of nHA crystallites dispersed in Dex-CS/nHA composite scaffold.

Samples	L (nm)	a = b (A°)	c (A°)	c/a	I_(002)_/I_(300)_
nHA	22.38	9.425	6.885	0.731	0.579
B	3	—	—	—	—
C	32.68	9.443	6.895	0.730	0.609
D	22.59	9.449	6.916	0.732	0.752

Lattice parameters (a and c) have been calculated from the diffraction planes (300) and (002), respectively, using the typical HCP unit cell plane spacing relationship^17^:

3 $$\frac{1}{{{\rm{d}}}^{2}}=\frac{4}{3}(\frac{{{\rm{h}}}^{2}+{\rm{hk}}+{{\rm{k}}}^{2}}{{{\rm{a}}}^{2}})+\frac{{{\rm{l}}}^{2}}{{{\rm{c}}}^{2}}$$

where d is the distance between neighboring planes in the set of Miller indices (hkl). The obtained values of the lattice parameters and c/a ratio are listed in Table 1. The obtained results reveal that there is a variation in the lattice parameters values of hydroxyapatite present in biopolymer/hydroxyapatite nanocomposites in comparison with pure hydroxyapatite. The biomimetic hydroxyapatite displayed analogous behavior in all nanocomposites, i.e., positive shift (elongation) was found along c-axis and a-axis (a = b). The ratio (R) of peak intensity of the planes (002) and (300), R_002/300_ = I_(002)_/I_(300)_, for the Dex-CS/nHA composites, is larger than that of the pure nHA as shown in Table 1. This means that the nHA crystals in the Dex-CS/nHA composites grow along the c-axis.

### Fourier transform infrared spectroscopy (FTIR)

**Figure 2:**
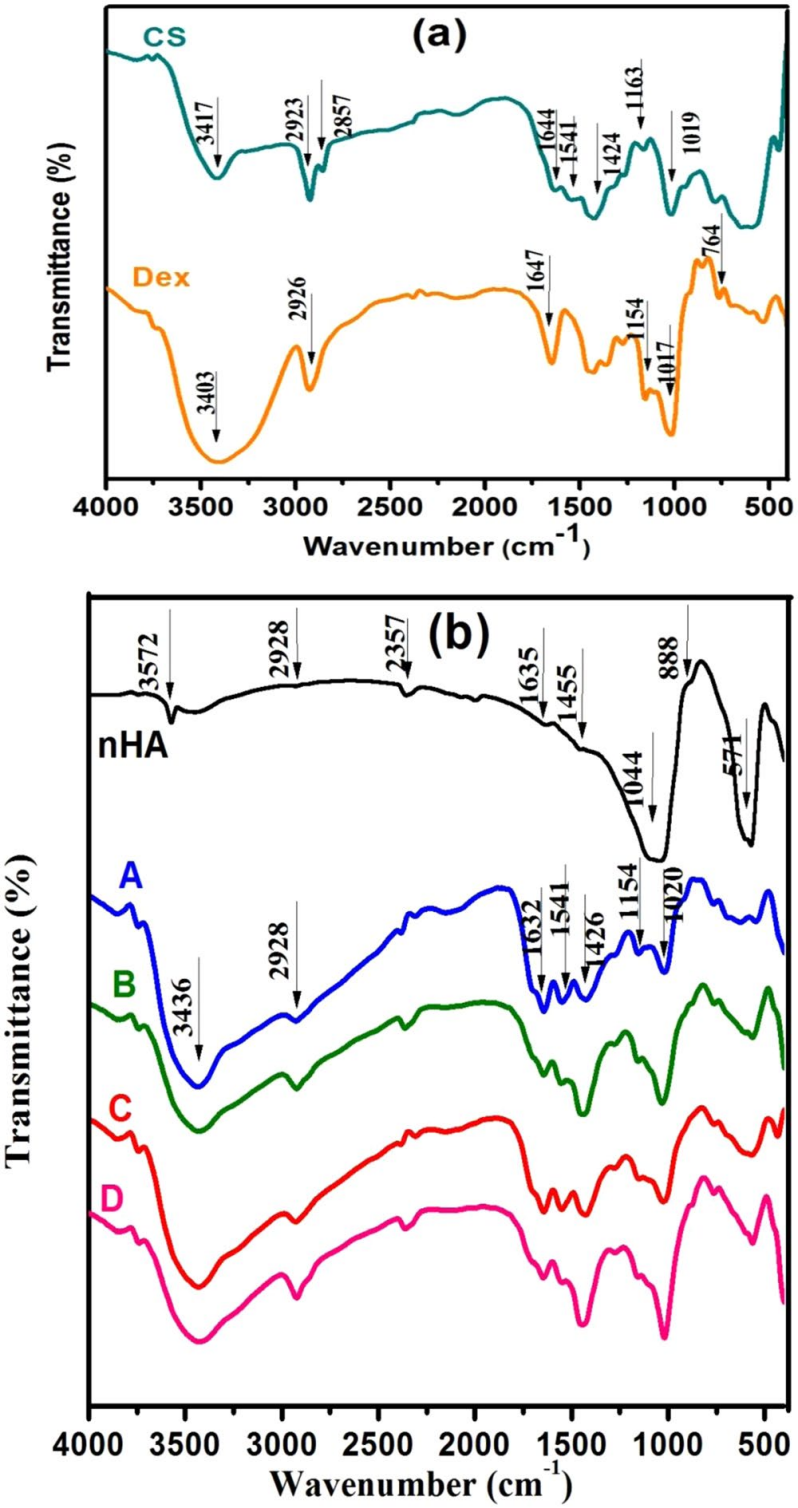
FT-IR spectra of (**a**) Dex and CS. (**b**) Dex-CS/nHA scaffolds with different contents of HA (A) 0, (B) 20, (C) 30 and (D) 40 Wt%.

The FTIR spectral investigation for Dex, CS, nHA and composition scaffolds (A, B, C and D) is shown in Fig. 2. The spectra elucidate the position of the functional groups in the prepared samples, their nature in terms of monomeric units and their linkages. The spectrum of pure nHA indicates the presence of clear OH^−^ band at 3572 cm^−1^, followed by some broad bands between 2928 and 2000 cm^−1^ that may correspond to HPO_4_^2−^ groups as mentioned before in the literature^18^. The shoulder at 1635 cm^−1^ for adsorbed water, evidenced the water absorption due to the high specific surface area of precipitated powders^19^. The shoulders at 1455 and 888 cm^−1^ are resulting from carbonate ions in apatite. The phosphate stretching vibration bands are designated at 1000–1100 cm^−1^ whereas the phosphate bending vibration bands situated at 500–600 cm^−1 ^^16^.

The FTIR spectrum of Dex-CS (composite scaffold A) reveals the complex formation by (i) the appearance of a small band at 2928 cm^−1^ which can be ascribed to the superposition of both polysaccharides (C–H) bands for Dex (at 2926 cm^−1^) and CS (at 2923 cm^−1^). (ii) there is a red shift of amide I band of CS at 1644 to 1632 cm^−1^ in the complex. This implies that there is a hydrogen bond between amide band groups of chitosan and hydroxyl groups of dextran^20^. (iii) the intensity of the band at 3436 cm^−1^ is enlarged owing to the overlapping between Dex(OH^−^) group and CS(N-H) group. (iv) the superposition of both (C-O) bonds for Dex and CS at 1020 cm^−1^ and the intensity increases in the complex. (v) The intensity of the two bands at 1541 and 1424 cm^−1^ decreased and shifted to higher frequency side to 1550 and 1426 cm^−1^ in the complex respectively. (vi) the band at 1154 cm^−1^ of Dex appeared at the same wavelength but its intensity increases in the complex.(vii) The band due to free acetic acid (1706 cm^−1^) is not recognized^21^.

The spectra of Dex-CS/nHA composite scaffolds with different proportions of nHA (20, 30 and 40 Wt%) exhibit characteristic bands typically present in both composite scaffold (A) and nHA. The increment of nHA percentage in the composite results in broadening of the band at 3436 cm^−1^ and its intensity increases due to the overlapping OH stretching band of nHA and Dex-CS composite^22^. In the composite scaffold D, the bands of amide I and amide II bending vibrations shifted to lower wave numbers at 1641 and 1542 cm^−1^, respectively, signifying chemical interaction between the amino groups of CS and the Ca^2+^ in the nHA or between the amino groups and PO_4_^3– ^^23^. The stretching vibrations of C-H band of Dex-CS shifted from 2928 cm^−1^ to 2923 cm^−1^ in Dex-CS/nHA with decreasing in its intensity by increasing nHA percentage. Figure 2 also shows that by enlarging HA content, the typical band of PO_4_ at 500–700 cm^−1^ became stronger^24^. Meanwhile, the wavelength and intensity of the other bands of Dex-CS changed slightly with no significant trend. The absorption bands are given in Table 2.

**Table 2:** Assignment of FTIR spectra of nHA, Dex-CS and Dex-CS/nHA composites presented in Fig. 3.

Samples	IR absorption bands (cm^−1^)	Description
nHA	3572	ʋ (O–H) for OH^−^ on the lattice sites of the nHA crystals
	1044	ʋ (P–O) for PO_4_^3−^
	600, 571	δ (P–O) for PO_4_^3−^
A	3436	δ (O–H)of Dex and ʋ (N–H) of CS
	2928	ʋ (C–H)
	1632	ʋ (–C=O–) Amide I
	1550	δ (N–H) in amide group
	1426	δ (C–H)
	1154, 1020, 765	ʋ(C–O–C)
B	3432	δ (O–H)of Dex and ʋ (N–H) of CS
	2924	ʋ (C–H)
	1646	ʋ (–C=O–) Amide I
	1555	δ (N–H) in amide group
	1156, 1031, 761	ʋ(C–O–C)
	601, 563	δ (P–O) for PO_4_^3−^
C	3432	δ (O–H)of Dex and ʋ (N–H) of CS
	2928	ʋ (C–H)
	1644	ʋ (–C=O–) Amide I
	1551	δ (N–H) in amide group
	1151, 1025, 762	ʋ(C–O–C)
	568	δ (P–O) for PO_4_^3−^
D	3427	δ (O–H)of Dex and ʋ (N–H) of CS
	2923	ʋ (C–H)
	1647	ʋ (–C=O–) Amide I
	1542	δ (N–H) in amide group
	1156, 1020, 763	ʋ(C–O–C)
	563	δ (P–O) for PO_4_^3−^

### Scanning electron microscopy (SEM)

**Figure 3:**
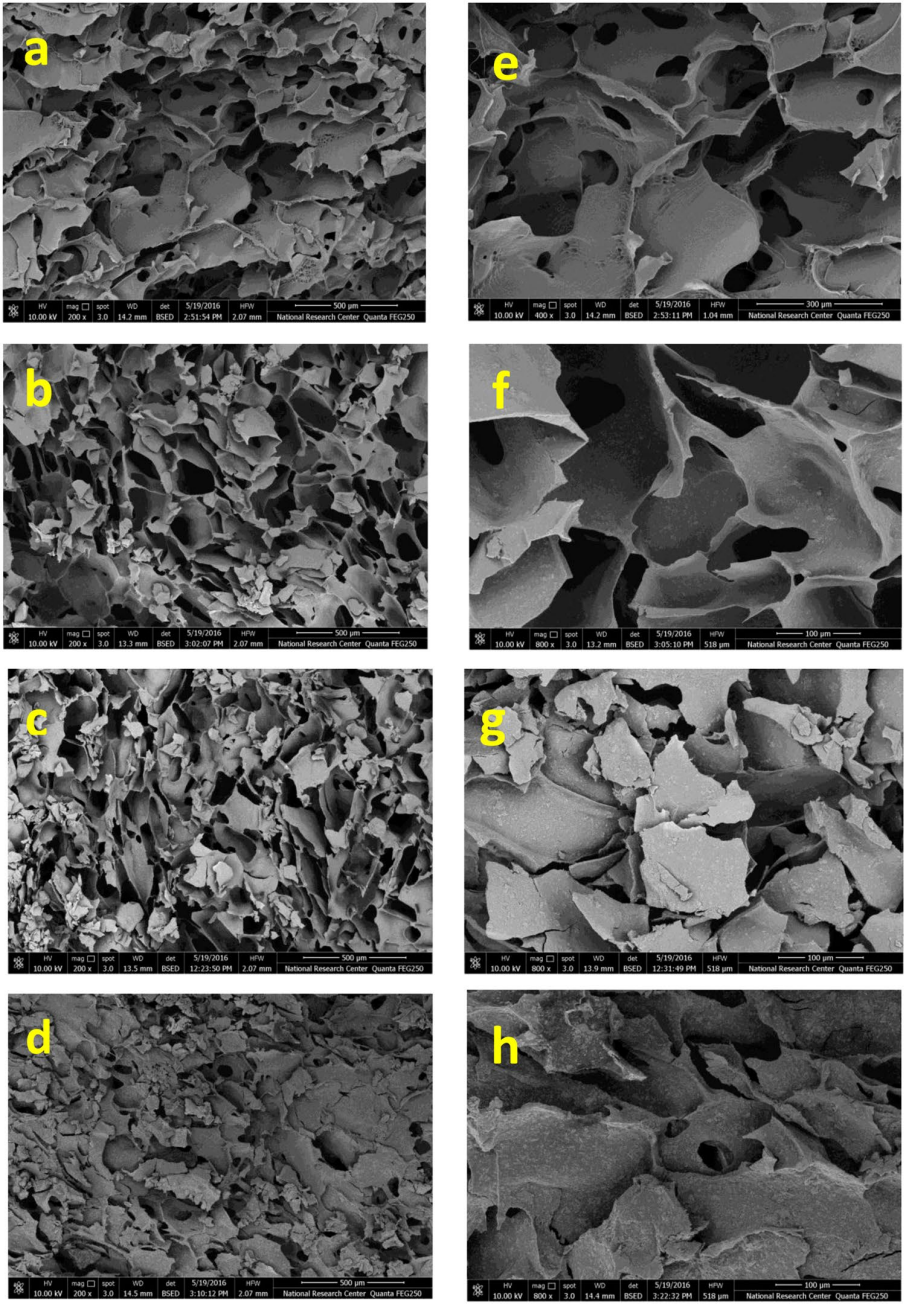
SEM micrographs of Dex-CS/nHA composite scaffolds with different nHA content at different magnifications (**a**,**e**) for 0 Wt%, (**b**,**f**) for 20 Wt%, (**c**,**g**) for 30 Wt% and (**d**,**h**) for 40 Wt%.

The SEM images of the composite scaffolds (A, B, C and D) are shown in Fig. 3. It could be observed that all scaffolds exhibit porous structures with good interconnection which directly influence the biological performance of materials. The cause of the porous morphology is that in the frozen process ceramic particles are extruded into gaps among ice crystals^25^. The high magnification SEM micrographs of the composite scaffolds are displayed in Fig. 3e–h. Many nano-HA particles have been observed to be uniformly dispersed in the polymer matrix, including the pore wall.

Then by adding nHA, the surface becomes rougher. All composite scaffolds are similar in their macroscopic morphology, which indicate that the addition of HA to system is not affect the porous structure; however, the microscopic morphology on pore wall surfaces is quite different because of different crystals created. It is clearly seen that the surface morphology of the scaffolds significantly altered by adding nHA, where the surface of the pure Dex-CS composite scaffold appeared smooth.

**Figure 4:**
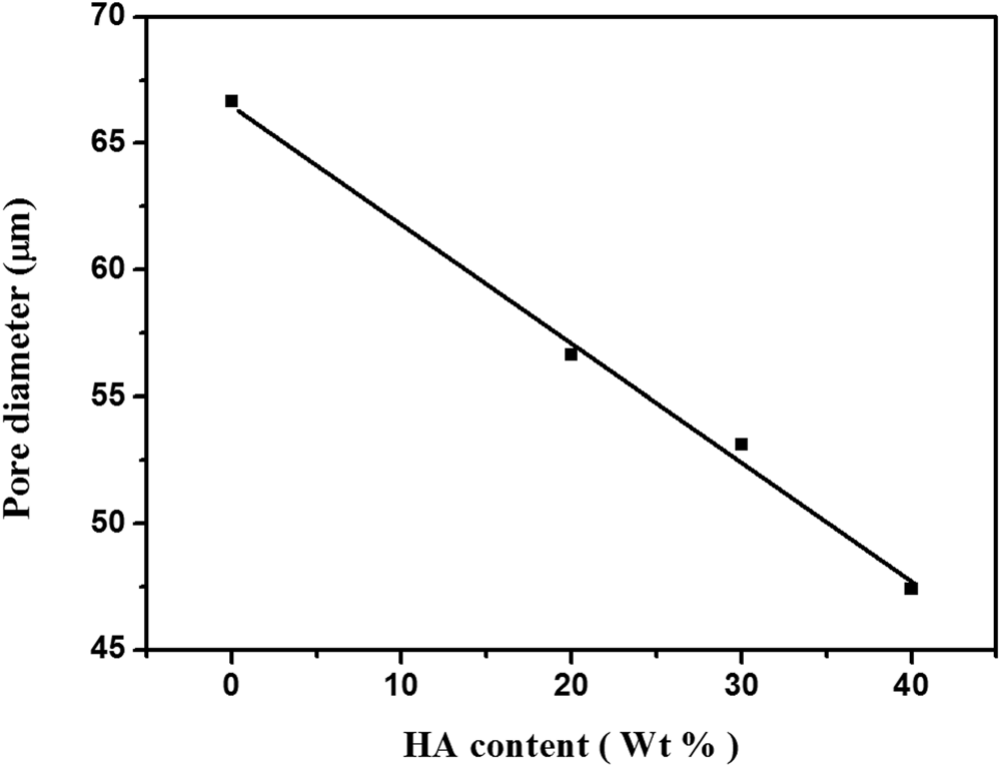
Pore diameter of Dex-CS/nHA composite scaffolds with different HA contents.

Comparing the four scaffolds, it could be noticed that the pore diameter of the scaffold decreases with increasing HA content as shown in Fig. 4. The average pore diameters have been estimated by analyzing scaffolds SEM images and were tabulated in Table 3. Also, it was observed that the incorporation of HA nanoparticles into the Dex-CS composite scaffolds decreased the porosity which is confirmed by the porosity assessment results. Moreover, the number of SEM images on scaffolds implied that HA nanoparticles collapsed the pore structure which is in convenient with that earlier discussed by Jin *et al*.^26^.

**Table 3:** Pore diameter, Density, percentage porosity and mechanical properties of the composites produced with various nHA concentrations.

Type of scaffold	Pore diameter (μm)	Density (g/cm^3^)	Porosity (%)	Young’s modulus (Mpa)	Compressive strength (Mpa)
A	66.7	0.809	82.9	0.016	0.18
B	56.6	0.810	71.6	0.027	0.58
C	53.1	0.835	69.7	0.036	0.59
D	47.4	0.838	67.5	0.042	0.63

From the above results of SEM, it could be found that the achieved composite scaffolds have good structural integrity and porosity. So, they are perfect candidates for tissue engineering and bone regeneration.

### Porosity and density

Density and porosity are important characteristics of the scaffolds which are related to each other. The apparent density of the scaffold can effect its mechanical strength, permeability, and existence of structural defects due to its porous nature^11^. Also, the porosity is a vital factor for a perfect scaffold to be used in tissue engineering applications^27^. Cells can mobile through the pores and become attached at appropriate positions in the scaffold for additional proliferation. Since a higher density of a scaffold generally leads to higher mechanical strength while a high porosity offers a promising biological environment, a balance between the porosity and density of a scaffold must be recognized for the specific application. The density of the composite scaffolds (A, B, C and D) has been measured, the obtained results ranges from 0.809 to 0.838 g/cm^3^ as shown in Table 3. These values are in good accordance with the apparent density of trabecular bone which ranges from 0.14 to 1.10 g/cm^3^. It is observed that, adding nHA to Dex-CS matrix increases the scaffold density. The increased density of the composite scaffold is ascribed to the greater material content in scaffold, as the water volume during the preparation of the scaffolds has been fixed. The enlarged density of the composite scaffold contributes to the better compressive performance.

**Figure 5:**
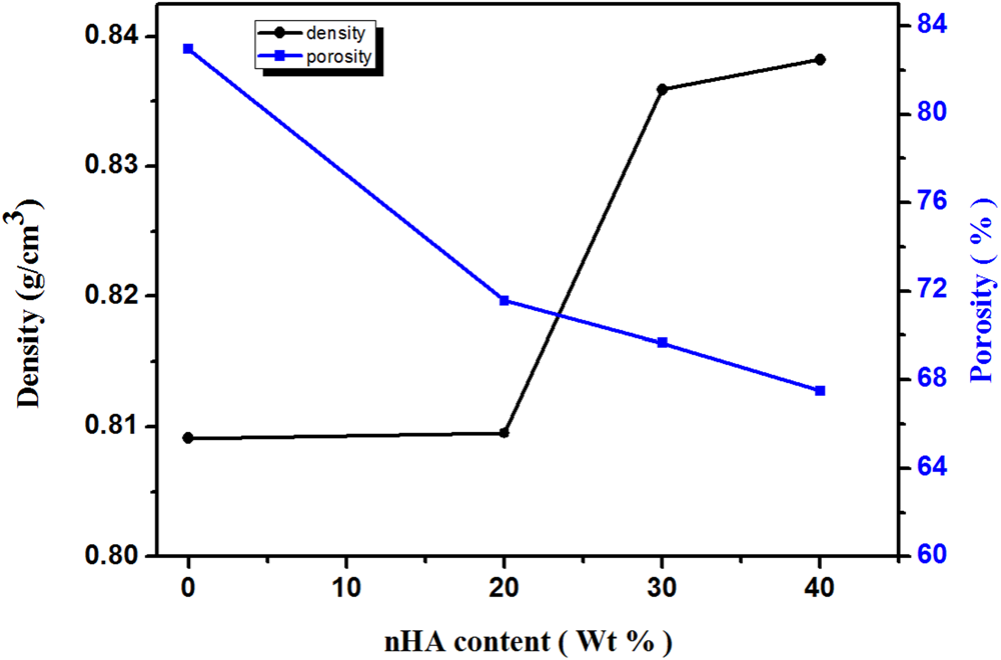
Density and percentage porosity of Dex-CS/nHA composite scaffolds with different HA contents.

The composites have a porosity range from 67.5 to 82.9% (Table 3 and Fig. 5), which is appropriate for bone regeneration, according to what was previously designated by Kruyt *et al*.^28^. The porosity of the composite decreased by increasing the nHA content due to the deposition of the nHA over the scaffold matrix and the intermolecular hydrogen bonding between -NH_2_ groups of chitosan and -OH groups of nHA, these results are similar to previous studies^29,30^. Other earlier researchers have found that the porosity decreased with adding ceramic particle content, since the increment in the density of the scaffold is confirmed^31^. The present nanocomposite scaffolds have sufficient porosity which helps in the passage of nutrients and oxygen to the inner regions of the composite scaffolds.

### Mechanical Properties

The scaffolds for bone tissue engineering should have sufficient mechanical properties for maintaining their integrity after implantation till new bone tissue regenerates and to transfer the load properly. The mechanical properties of freeze-dried scaffolds are usually tested by compression mode^32^. The influence of nHA on the mechanical properties of the Dex-CS scaffolds in dry state has been explored. The obtained values of Young’s modulus and compressive strength have been summarized in Table 3.

The linear variation of stress versus strain (up to the strain value of about 15%) was detected in the first phase of the compression investigation. This is the stage of elastic distortion and this linear stage of the curve was used to estimate the Young’s modulus of the composite. Within this stage of strain, the scaffolds distorted elastically and returned to their initial dimensions when the load was removed. After the linear stage of the curve, the scaffolds did not return to their initial dimensions when the load was removed and the starting of the destruction process was detected.

**Figure 6:**
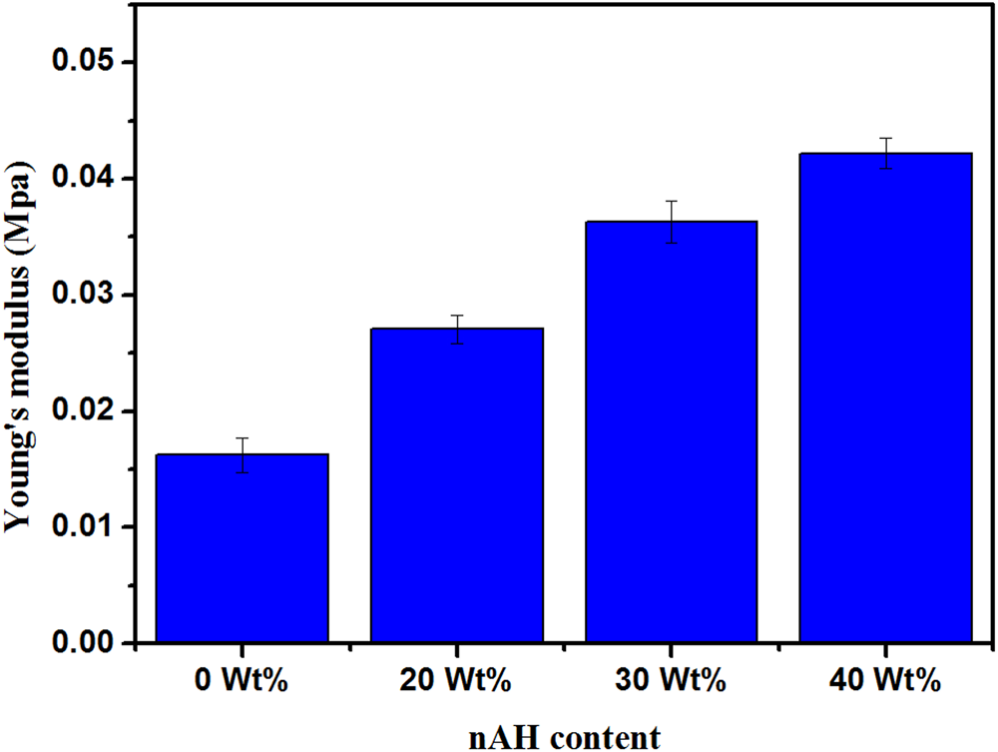
Young’s modulus of Dex-CS/nHA composite scaffolds with different HA contents.

**Figure 7:**
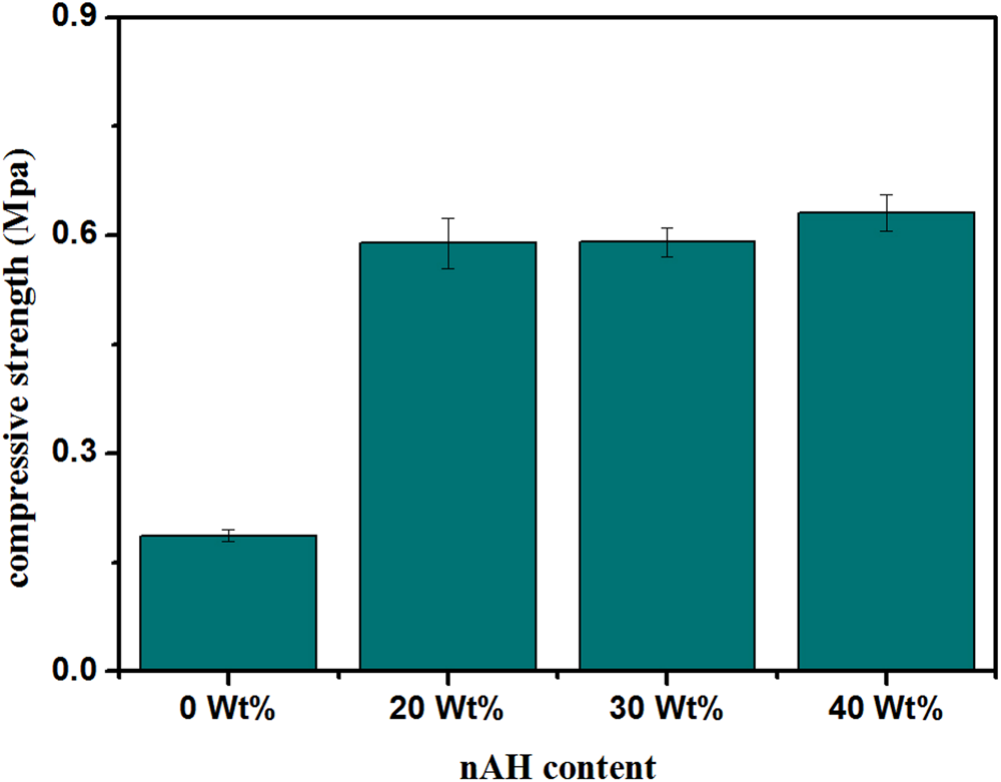
Compression strength of Dex-CS/nHA composite scaffolds with different HA contents.

As previously designated in literature, the polymers used for scaffold fabrication, such as chitosan and dextran, existing weak mechanical properties which limit their applicability in bone tissue engineering^33^. To overcome this handicap, the adding of calcium phosphate ceramics to chitosan has been described to enhance the mechanical properties of the scaffolds^33,34^. Therefore, as can be observed from Figs 6 and 7, addition of nHA particles to the Dex-CS matrix improves both Young’s modulus and compression strength.

The Young’s moduli of the prepared composites were moderately low and different from those of natural spongy bone (0.18–0.33 GPa)^35^ and compact bone (17–19 GPa)^36^. It could be proposed that the strain was taken by Dex/CS matrix while the ceramic granules were only slightly deformed through compression testing.

The increased compressive strength provides evidence that nHA interacted well within pore wall of CS matrix. Furthermore, these results confirm the excellent dispersion of nHA inside the polymer matrix has a remarkable influence on the improvement of the mechanical properties of the porous composite scaffolds. Also, the enlarged density and reduced porosity of the composite scaffold by totaling nHA contribute to the enhancement in the mechanical properties.

## Conclusions

In this study, a novel Dex-CS/nHA composite scaffolds with different HA contents (0, 20, 30 and 40 Wt%) were synthesized by freeze-drying method. The crystallite size of composite scaffold with HA content of 40 Wt% is ~23 nm, which is close to the crystallite size of bioapatite in bone tissue. The SEM results revealed that the scaffolds had an extremely interconnected porous structure which directly influences the biological performance of materials. As the HA content was regularly increased, the surface of the scaffold became rougher. The prepared nanocomposite scaffolds have sufficient porosity which helps in the passage of nutrients and oxygen to the internal regions of the composite scaffolds. The compressive strength of the composite scaffolds increases by increasing HA content. It can be established that the fabricated scaffolds had desirable physico-chemical properties to meet the essential requirement for bone tissue engineer materials.
